# Microfluidic converging/diverging channels optimised for homogeneous extensional deformation

**DOI:** 10.1063/1.4954814

**Published:** 2016-07-05

**Authors:** K. Zografos, F. Pimenta, M. A. Alves, M. S. N. Oliveira

**Affiliations:** 1 James Weir Fluids Laboratory, Department of Mechanical and Aerospace Engineering, University of Strathclyde, Glasgow G1 1XJ, United Kingdom; 2 Centro de Estudos de Fenómenos de Transporte, Departamento de Engenharia Química, Faculdade de Engenharia da Universidade do Porto, 4200-465 Porto, Portugal

## Abstract

In this work, we optimise microfluidic converging/diverging geometries in order to produce constant strain-rates along the centreline of the flow, for performing studies under homogeneous extension. The design is examined for both two-dimensional and three-dimensional flows where the effects of aspect ratio and dimensionless contraction length are investigated. Initially, pressure driven flows of Newtonian fluids under creeping flow conditions are considered, which is a reasonable approximation in microfluidics, and the limits of the applicability of the design in terms of Reynolds numbers are investigated. The optimised geometry is then used for studying the flow of viscoelastic fluids and the practical limitations in terms of Weissenberg number are reported. Furthermore, the optimisation strategy is also applied for electro-osmotic driven flows, where the development of a plug-like velocity profile allows for a wider region of homogeneous extensional deformation in the flow field.

## I. INTRODUCTION

A large amount of industrial processes and scientific investigations dealing with Newtonian and non-Newtonian fluids are characterised by the occurrence of strong extensional flows. There is a current demand for appropriate devices capable of assisting in the investigation of the extensional behaviour and the characterisation of extensional material properties of fluids of interest, in particular, those exhibiting complex rheological behaviour such as polymer solutions or various biofluids (Galindo-Rosales *et al*., 2013 and Haward, 2016). Unlike Newtonian fluid flows—in which the extensional viscosity is proportional to the shear viscosity (Trouton ratios of 3 or 4 for uniaxial and planar extension, respectively)—viscoelastic fluid flows often lead to significantly larger flow resistance due to strong extensionally thickening effects, with Trouton ratios that can be orders of magnitude greater than for Newtonian fluids (Haward, 2016). This makes thorough experimental characterisation of extensional properties of viscoelastic fluids crucial in various contexts, ranging from fundamental studies to industrial applications, aiming to: accurately describe and predict their behaviour; effectively control their flow; design efficient and safe devices/fluidic components; detect subtle dissimilarities in their composition (e.g., for product quality control); provide quality-assurance of final products (e.g., in polymer or food processing industries).

Lab-on-a-chip platforms have been proven a very powerful tool in the context of extensional flows of complex fluids (Rodd *et al.*, 2005 and Galindo-Rosales *et al*., 2013). The characteristic small length scales (1 *μ*m–1000 *μ*m) of microfluidic devices allow the generation of large deformations and deformation rates for relatively small flow rates, enhancing mechanical properties that might otherwise be masked by inertial effects in macro-scale flows. The small amount of sample needed to operate the microfluidic devices and their ability to reproduce precisely controlled, three dimensional environments, make them a promising candidate over other techniques used conventionally in biomedical research (Sackmann *et al*., 2014 and Sousa *et al.*, 2016). Examples include studies of cell responses, molecular stretching, as well as droplet deformation and other interfacial studies (Shui *et al*., 2007; Velve-Casquillas *et al.*, 2010; Mulligan and Rothstein, 2011; Mai *et al*., 2012; and Gossett *et al.*, 2012). Microfluidics have also found a *niche* application for investigating and characterising the rheological behaviour of viscoelastic fluids (Pipe and McKinley, 2009; Galindo-Rosales *et al*., 2013; and Haward, 2016), both under shear and extensional deformation.

Abrupt contractions are arguably the most frequently used geometries for studying extensional flows. Despite their geometric simplicity, they are able to produce flows with a combination of strong shear effects close to the walls and strong extensional effects along the centreline region in the vicinity of the contraction (Rothenstein and McKinley, 2001). Such entry flows have been established as one of the most appropriate geometries for benchmarking the efficiency of computational methods for non-Newtonian fluids (Hassager, 1988; Owens and Phillips, 2002; and Alves *et al*., 2003b) and have been extensively used experimentally for investigating the mechanisms of fluid elasticity (Boger, 1987; Rothenstein and McKinley, 2001; and Rodd *et al.*, 2005; 2007), where the “excess” pressure drop due to extensional flow in the contraction is correlated to important viscoelastic normal-stress effects (Ober *et al.*, 2013). However, abrupt contractions fail to produce homogeneous extension conditions and therefore are unlikely to establish a region of constant strain-rate (Oliveira *et al.*, 2008). As with shear viscosity measurements, where shear rheometers generate a constant shear rate canonical flow, which allows the measurement of the shear viscosity as a function of shear rate, for an extensional rheometer constant extension rate would be ideal for investigating extensional properties of the fluids (Galindo-Rosales *et al*., 2013). With that goal in mind, Alves (2008) introduced the “peculiar” shape of an optimised cross-slot, named OSCER (Optimised Shape Cross-slot Extensional Rheometer), by demonstrating numerically its ability to generate homogeneous extension along the centrelines of the flow field for both Newtonian and viscoelastic fluids. Haward *et al.* (2012) fabricated and studied experimentally the performance of the optimised cross-slot, demonstrating the good performance of the OSCER device for both Newtonian and low viscosity polymer solutions, validating its potential for extensional rheology measurements. The same configuration has been later employed for investigating the rheological properties of hyaluronic acid (Haward *et al.*, 2013).

Galindo-Rosales *et al*. (2013) reviewed various micro-fabricated configurations for potential use in experimental studies related to elongational flows and highlighted the relevance of microfluidics in the context of extensional rheometry. Hyperbolic shaped microchannels were among the geometries suggested for this purpose, and have also been discussed in a recent review by Haward (2016). The idea of constrained converging flows was proposed by Cogswell (1978, 1972), in order to enforce elongation and assist in extensional flow rheological measurements of polymer melts. James *et al*. (1990) introduced the principles of a hyperbolic converging rheometer pointing out its advantage to generate constant strain-rates along the centreline of the flow. Compared to configurations like the OSCER device, the advantage of this type of entry flow geometry is its intrinsic simplicity, with only one inlet and one outlet. In this case, the nominal strain-rate can be controlled by varying the volumetric flow rate of a single stream (instead of at least three streams which are required for cross-slot and flow focusing devices), making it very practical for experimental studies. To the best of our knowledge, Oliveira *et al.* (2007) were among the first to consider a micro-fabricated hyperbolic configuration as a potential microfluidic rheometer and studied its performance both numerically and experimentally, using a Newtonian fluid. They presented a detailed study of the flow kinematics in a hyperbolic contraction followed by an abrupt expansion and pointed out the difficulty in distinguishing extensional from shearing effects within the contraction region, with the flow being non-homogeneous and the developed strain-rate deviating from the ideal uniform profile. The same configuration was also used for estimating the apparent extensional viscosity of polyethylene oxide solutions (McKinley *et al.*, 2007), for investigating the flow of low viscosity Boger fluids (Campo-Deaño *et al.*, 2011) and also for mimicking flows along stenoses in the human micro-circulatory system using blood analogue solutions (Sousa *et al.*, 2011). Additionally, this type of converging/diverging geometries have been used to study the deformability of white blood cells (Rodrigues *et al.*, 2015) and of red blood cells under strong extensional flow, for potential use in diagnosis of blood diseases (Lee *et al*., 2009; Yaginuma *et al.*, 2013; and Wu and Feng, 2013). Ober *et al.* (2013) extended the study on the use of hyperbolic channels for rheological purposes by considering a micro-channel with a symmetric hyperbolic contraction/expansion used in the commercially available “Extensional Viscometer-Rheometer-On-a-Chip” (EVROC). The device includes four pressure sensors along the length of the channel for separately evaluating the pressure drop due to shear effects in the fully developed regions upstream and downstream of the contraction, where the flow is fully developed, and the pressure drop across the hyperbolic contraction/expansion. It was intended that by subtracting these two pressure drops, it would be possible to evaluate the extra pressure drop due to elastic normal stresses alone. However, they reported that the configuration used was producing a non-homogeneous flow field, with entrance and exit effects resulting in a region with combined shear and elongational characteristics. In the present work, we overcome the challenge of non-homogeneity of the velocity field by optimising the shape of a converging/diverging channel to generate the ideal strain-rate profile along the centreline of the flow for use in elongational studies of macromolecules (e.g., DNA) or cells and for potential use as an extensional rheometer.

An attractive advantage of microfluidic devices is that fluid flow within the channels can be achieved efficiently in different ways, including *pressure-driven* or *electro-osmotic* flows (Webster *et al*., 2011). For a pressure-driven flow, the fluid motion is frequently imposed using syringe pumps or pressure pumps, resulting in a variety of fully developed velocity profiles depending on the cross-sectional aspect ratio, as a consequence of fluid-wall interactions, and on the rheology of the fluid. For an electro-osmotic flow (EOF), the charged walls of the microfluidic channel attract the counterions of the fluid and form an electric double layer (EDL) near the interface. By applying an electric field between the inlet and the outlet of the microchannel, the electrically neutral bulk is set in motion due to the electric force acting on the EDL, generating a plug-like velocity profile. Pressure-driven flows are usually the most common, but the velocity profile dependence on the channel position can be undesirable for some applications (Galindo-Rosales *et al*., 2014). The typical plug-like flow of electro-osmosis could reduce this effect by extending the extensional behaviour along the centreline over a wide region, and has found many applications in engineering, biomedicine, and chemistry (Wang *et al.*, 2009). In this work, we perform optimisations for both pressure-driven and EOF devices.

The remainder of the paper is organised as follows: The characteristic dimensions of the configuration studied are given in Sec. II, together with the ideal velocity and strain-rate profiles that are used as targets in the optimisation procedure. Sec. III presents the optimisation strategy followed for finding the optimal shape of each configuration, and Sec. IV presents the governing equations of fluid motion for pressure driven and electro-osmotic flows. The optimisation of pressure driven flows is examined in Sec. V where we discuss the effects of the contraction length, the channel depth and assess the limits of the designs for increasing Reynolds (Re) and Weissenberg (Wi) numbers. Electro-osmotic flows are considered in Sec. VI, and the optimal shape solutions are presented for various geometry aspect ratios. Finally, the main conclusions of this study are summarised in Sec. VII.

## II. GEOMETRY DEFINITION

The primary aim of this work is to find the optimised shape of a converging/diverging channel that is able to produce wide regions of constant strain-rate, $\dot{\varepsilon }=\partial u/\partial x$, along the centreline of the flow for pressure-driven and electro-osmotic flows.

**FIG. 1.**
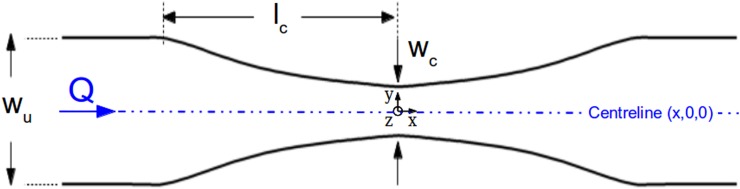
Configuration of the converging/diverging geometry.

**FIG. 2.**
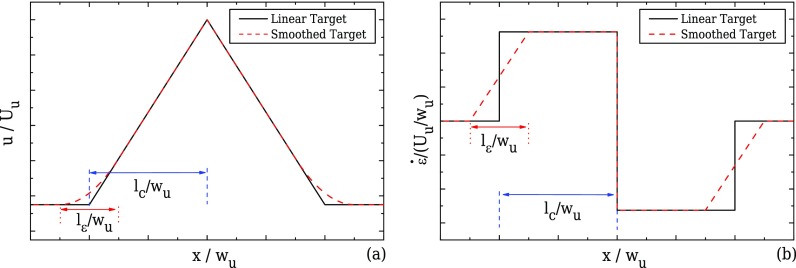
Ideal velocity (a) and strain-rate profiles (b) along the centreline of the flow.

The flow is driven from one inlet with an average velocity, *U_u_*, towards the outlet of the device as shown in Fig. 1. As the fluid flows through the contraction, the velocity along the centreline of the flow, *u*, will ideally start to increase linearly (James *et al*., 1990 and James, 1991) as shown in Fig. 2(a), reaching a maximum value at the throat of the contraction/expansion region. In the same manner, the fluid velocity is expected to decrease linearly in the symmetric diverging part. This ideal behaviour results in a region of strong extension, where the strain-rate remains constant along the centreline of the flow as shown in Fig. 2(b). The geometry is characterised by an upstream width, *w_u_*, and a contraction width, *w_c_* (cf. Fig. 1), which define the contraction ratio CR = *w_u_*/*w_c_*, the length of the contraction, *l_c_*, and produces a total extension, described by the value of Hencky strain, *ϵ_H_* = ln(CR). A device that is able to generate these ideal flow characteristics can be useful for extensional rheology as well as for performing single cell/droplet/molecule studies under homogeneous extensional flow, such as droplet deformation, DNA and actin filament stretching, among others.

In the converging part of the contraction, the lateral walls approach each other and create a narrow region where the fluid is stretched. Typically, the cross-sections of microfluidic platforms are not circular but exhibit a rectangular shape with constant depth. As such, the channel aspect ratio varies significantly along the streamwise direction within the contraction region. This, together with typical abrupt or short-length hyperbolic configurations used (Oliveira *et al.*, 2007 and Ober *et al.*, 2013), leads to non-ideal flow kinematics resulting in a non-homogeneous strain-rate along the centreline. In this work, we attempt to overcome this problem by employing optimisation techniques that change the shape of the microchannel in order to approach the ideal profiles illustrated in Fig. 2. The choice of a symmetric converging/diverging contraction is based on the fact that this configuration provides a constant strain-rate along the entire length of the contraction/expansion, with a positive strain-rate in the converging region and a negative value in the expansion region, where the stretching and relaxation processes can be analysed under homogeneous flow conditions. On the other hand, a smooth contraction/abrupt expansion configuration similar to Oliveira *et al.* (2007) would generate a large undershoot of the strain-rate in the vicinity of the expansion plane, due to the sudden decrease in the velocity along the centreline, which would not be ideal for devices intended to produce homogeneous extension.

We consider geometries with an upstream width eight times larger than the contraction width (CR = 8). For all the cases studied, the contraction length is correlated to the upstream width with the use of a factor *n*_1_, such that *l_c_* = *n*_1_*w_u_*. The effect of using different contraction lengths on the final optimised designs are reported in Section V B, highlighting the importance of this choice. In the case of three dimensional geometries (3D), we found that the choice of the depth of the device affects significantly the final shape, as shown in the results presented in Section V C.

The envisioned ideal flow field in the converging/diverging geometries corresponds to a linear velocity profile along the centreline. However, this profile imposes instantaneous step changes in the strain-rate at the beginning and at the end of the contraction, as illustrated in Fig. 2(b). This limiting behaviour is not possible in reality because the gradient of the velocity profile is a continuous function and therefore we consider a smooth transition in the velocity profile that is first order differentiable and which yields a linear transition in the strain-rate profile (cf. Fig. 2(b)) except at the throat, *x*/*w_u_* = 0. The performance of the abrupt transition profile was also examined and more information can be found in the supplementary materials provided.

The general form of the target velocity profile is given in Eq. (1) and holds for both 2D and 3D geometries. It considers a smooth transition of the velocity when the fluid enters the converging part and exits the diverging part of the channel. The smoothing of the target profile is achieved by employing a region of length *l_ε_*, which is correlated to the upstream width by the use of a factor *n*_2_, such that *l_ε_* = *n*_2_*w_u_*. For all the cases studied here, we considered *n*_2_ = 1. In this transition region, the velocity is expressed by a second-order polynomial as shown in Fig. 2(a).

$$\tilde{u}=\{\begin{array}{cc} {\tilde{u}}_{u} & \;\text{if}\;\tilde{x}\leq -n_{1}-\frac{n_{2}}{2}\\ f_{2}{\left[\tilde{x}+n_{1}+\frac{n_{2}}{2}\right]}^{2}+{\tilde{u}}_{u} & \;\text{if}\;-n_{1}-\frac{n_{2}}{2}<\tilde{x}<-n_{1}+\frac{n_{2}}{2}\\ f_{1}\tilde{x}+{\tilde{u}}_{c} & \;\text{if}\;-n_{1}+\frac{n_{2}}{2}\leq \tilde{x}\leq 0\\ -f_{1}\tilde{x}+{\tilde{u}}_{c} & \;\text{if}\;0\leq \tilde{x}\leq n_{1}-\frac{n_{2}}{2}\\ f_{2}{\left[\tilde{x}-n_{1}-\frac{n_{2}}{2}\right]}^{2}+{\tilde{u}}_{u} & \text{if}\;n_{1}-\frac{n_{2}}{2}<\tilde{x}<n_{1}+\frac{n_{2}}{2}\\ {\tilde{u}}_{u} & \;\text{if}\;\tilde{x}\geq n_{1}+\frac{n_{2}}{2}. \end{array}$$ (1)

All symbols with tilde represent normalised values, such that $\tilde{x}=x/w_{u},\;\tilde{u}=u/U_{u}$, where *U_u_* is the average upstream velocity, and the dimensionless parameters *f*_1_ and *f*_2_ are given by $f_{1}=({\tilde{u}}_{c}-{\tilde{u}}_{u})/n_{1}$ and $f_{2}=({\tilde{u}}_{c}-{\tilde{u}}_{u})/2n_{1}n_{2}$, respectively. When no smoothing is desired (*n*_2_ = 0; *l_ε_* = 0), the intervals of the smoothed function drop to zero, yielding only the linear velocity profile. The resulting normalised strain-rate profiles corresponding to Eq. (1) are given by ($\dot{\varepsilon }=\partial u/\partial x$)

$$\dot{\varepsilon }/(U_{u}/w_{u})=\{\begin{array}{cc} 0 & \;\text{if}\;\tilde{x}\leq -n_{1}-\frac{n_{2}}{2}\\ 2f_{2}[\tilde{x}+n_{1}+\frac{n_{2}}{2}] & \;\text{if}\;-n_{1}-\frac{n_{2}}{2}<\tilde{x}<-n_{1}+\frac{n_{2}}{2}\\ f_{1} & \;\text{if}\;-n_{1}+\frac{n_{2}}{2}\leq \tilde{x}\leq 0\\ -f_{1} & \;\text{if}\;0\leq \tilde{x}\leq n_{1}-\frac{n_{2}}{2}\\ 2f_{2}[\tilde{x}-n_{1}-\frac{n_{2}}{2}] & \text{if}\;n_{1}-\frac{n_{2}}{2}<\tilde{x}<n_{1}+\frac{n_{2}}{2}\\ 0 & \;\text{if}\;\tilde{x}\geq n_{1}+\frac{n_{2}}{2}. \end{array}$$ (2)

Figure 2(b) shows that the smooth target velocity profile of Eq. (1) produces a linear increase/decrease in the strain-rate along the centreline of the flow at the beginning/end of the contraction/expansion region, instead of the step profile of the linear velocity profile. At the contraction throat ($\tilde{x}=0$), there is a discontinuity in the strain-rate profile, but given the small total width of the channel, the target profile is reasonably well approximated as will be shown.

## III. OPTIMISATION STRATEGY

**FIG. 3.**
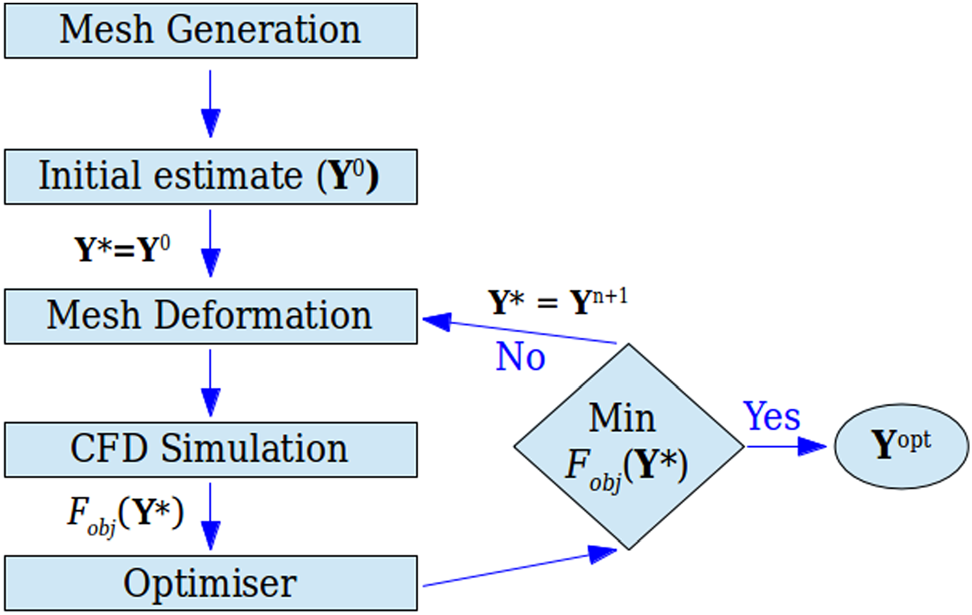
Flow chart of the optimisation procedure.

The optimisation procedure is described schematically in the flow chart of Fig. 3. An iterative procedure combining an automatic mesh generation routine and a fluid flow solver coupled with an optimiser allows us to determine numerically the appropriate boundary shape of the device for a prescribed flow field, such as Eq. (1).

The outcome of each computational fluid dynamics (CFD) simulation from every set $Y^{*}$ represents a single solution of a general unknown *objective* function. Here, we define the value of the objective function as a cell-averaged velocity difference evaluation between the ideal behaviour and the CFD results

$$F_{obj}=\sum_{i}\left|{\tilde{u}}_{i}-{\tilde{u}}_{target,i}\right|\Delta {\tilde{x}}_{i},$$ (3)

where ${\tilde{u}}_{target,i}$ is the desired dimensionless velocity value in each computational cell *i* required to obtain the ideal velocity profile described by Eq. (1) and shown in Fig. 2(a); ${\tilde{u}}_{i}$ is the dimensionless velocity evaluated from the CFD solver at each *i*-cell along the centreline of the flow; and $\Delta {\tilde{x}}_{i}$ is the streamwise dimensionless spacing of the computational cell *i*. This optimisation procedure is characterised by its non-linearity and therefore is not easy to solve. In this work, we employ two freely available derivative-free optimisers, NOMAD (Le Digabel, 2011; Audet and Dennis, Jr., 2006; and Audet *et al*., 2009) and CONDOR (Berghen and Bersini, 2005), appropriate for performing constrained optimisations. NOMAD optimiser is based on the Mesh Adaptive Direct Search algorithm whereas CONDOR is a generalisation of Powell's UOBYQA methodology (Powell, 2002), developed to deal with non-linear constrained optimisation problems.

As indicated in Fig. 3, an initial estimate $Y^{0}$ of the design points is given as input to the mesh generation program for creating the discretised geometry. Here, two different mesh deformation procedures have been used, considering 12 equally distributed design points along the flow direction. One is based on the geometrical deformation of an object using Non-Uniform Rational B-Splines (NURBS, Lasmunsin and Waggenspack, 1994) and was used in the optimisations presented in Sec. V, whereas the second method uses Catmull-Rom interpolating splines (Catmull and Rom, 1974), to generate the shape of the geometries discussed in Sec. VI. After the geometry is generated/deformed, the flow solver computes the corresponding flow field, from which the value of a single objective function, *F_obj_*, is calculated. The current $F_{obj}(Y^{*})$ is then examined by the optimiser and a new set $Y^{n+1}$ is produced by the optimiser, which is used to generate a new geometry. The aim of the optimiser is to minimise the value of the objective function, approximating the desired velocity profile by minimising Eq. (3), ideally for a small number of *F_obj_* evaluations. When a minimum of *F_obj_* is approached, the final optimised solution $Y^{opt}$ is obtained. We note that the optimisers used in this work do not guarantee that the global optimum solution is always achieved, since a local minimum can be found. However, different initial estimates $Y^{0}$ allow to obtain good results.

## IV. GOVERNING EQUATIONS

The CFD simulations performed for each evaluation of the objective function consider a laminar, incompressible, and isothermal fluid flow, solving numerically the continuity and momentum equations

∇·u=0, (4)

$$\rho \left(\frac{\partial u}{\partial t}+u\cdot \nabla u\right)=-\nabla p+\nabla \cdot \tau +F,$$ (5)

where *ρ* is the fluid density, **u** is the velocity vector, *p* is the pressure, τ is the extra-stress tensor, and **F** is the electric body force per unit volume which is required to simulate electro-osmotic flows. For pressure-driven flows, **F** = **0**. In the optimisations, we consider creeping flow conditions (Re → 0), a good approximation in microfluidics. Therefore, with the exception of a small number of CFD simulations presented in Sec. V, the convective term in the momentum equation is neglected. Initially, we search for a general design that exhibits the ideal flow kinematics using Newtonian fluids and then we investigate the operational limits of the optimised geometries in terms of Re for Newtonian fluids, and Wi for viscoelastic fluids. The Weissenberg number is here defined as $\text{Wi}=\lambda (U_{c}-U_{u})/l_{c}$, where *λ* is the fluid relaxation time. The Reynolds number is defined as $\text{Re}=\rho U_{u}Dh_{u}/\eta_{0}$, where $\eta_{0}=\eta_{s}+\eta_{p}$ is the total zero shear viscosity, *η_p_* the polymer viscosity (*η_p_* = 0 for Newtonian fluids), *η_s_* the solvent viscosity, and $Dh_{u}$ the upstream hydraulic diameter defined as $Dh_{u}=2w_{u}d/(w_{u}+d)$, where *d* is the depth of the device.

For viscoelastic fluid flow, two models were tested, namely, the Oldroyd-B and the linear form of the PTT model (Phan-Thien and Tanner, 1977). The Oldroyd-B model is used to assess the response of viscoelastic fluids with constant shear viscosity, whereas the PTT model was used because of its additional ability to predict shear-thinning behaviour. Both models can be expressed by the compact form of the simplified Phan-Thien and Tanner constitutive equation

$$\lambda \overset{\Delta }{\tau_{p}}+f(\tau_{p})\tau_{p}=\eta_{p}(\nabla u+\nabla u^{T}),$$ (6)

where $\overset{\Delta }{\tau_{p}}$ is the upper-convected derivative of the polymeric component of the extra-stress tensor, $\tau_{p}$. The stress function, $f(\tau_{p})$, is expressed as a linear function of the trace of the polymeric stress tensor, $\text{Tr}(\tau_{p})$

$$f(\tau_{p})=1+\frac{\lambda \varepsilon }{{\mathrm{\eta \backslash neth}}_{p}}\text{Tr}(\tau_{p}),$$ (7)

where *ε* is the extensibility parameter that affects the elongational properties of the fluid and sets an upper bound for the extensional viscosity (Phan-Thien and Tanner, 1977; Oliveira and Pinho, 1999; and Alves *et al*., 2001). When *ε* = 0, the Oldroyd-B model is recovered and the extensional viscosity is unbounded. For the viscoelastic cases, the extra-stress tensor in the momentum equations is decomposed in two parts, the solvent and the polymeric components

$$\tau =\eta_{s}(\nabla u+\nabla u^{T})+\tau_{p}.$$ (8)

Additionally, for both viscoelastic models, the ratio of the solvent viscosity, *η_s_*, to the total zero shear viscosity, *η*_0_, known as solvent viscosity ratio, *β*, needs to be defined. In the PTT model, we consider *β* = 0.01 and *ε* = 0.25, which are typical values for concentrated polymer melts, whereas for the Oldroyd-B model the viscosity ratio was set at *β* = 0.50 representative of a constant-viscosity Boger fluid.

For pressure driven flow simulations (Sec. V), the electric force, **F**, is zero, and the fluid flow is solved using an in-house implicit finite-volume CFD solver, developed for collocated meshes, which is described in detail in Oliveira *et al*. (1998) and Oliveira (2001). The coupling of pressure and velocity fields is achieved using the SIMPLEC algorithm for collocated meshes with the Rhie and Chow (1983) interpolation technique. The convective terms in the momentum and constitutive equations are discretised using the CUBISTA high-resolution scheme (Alves *et al*., 2003a), while the diffusive terms are discretised with central differences. The transient term in the momentum and constitutive equations are evaluated using a first-order implicit Euler scheme. We note that since we are concerned with steady-state solutions, the lower order of accuracy of the transient term is irrelevant, as this term vanishes when steady-state is approached.

In converging flow configurations, die walls converge to a narrow region in the middle of the contraction and thus are characterised by strong shear and elongational effects. Close to the walls the flow is shear dominated, along the centreline, it is strongly extensional, but the intermediate regions exhibit complex flow kinematics. This physical drawback may affect experimental results when controlled flow kinematics are required, and thus, it is of paramount importance for experimentalists to know the level of these interactions. In order to reduce shearing effects, we have also performed optimisations considering EOF. For electrokinetic flow, a plug-like velocity profile is obtained, thus reducing the shearing effects in the vicinity of the walls which allows for a wider region of extensional flow. When considering EOF, the electrical body force per unit volume, **F**, in Eq. (5) is expressed as

$$F=\rho_{e}E,$$ (9)

where **E** is the electric field and *ρ_e_* is the electric charge density. In this work, we consider the Debye-Hückel approximation for the electric charge density, which is expressed as (Bruus, 2008)

$$\rho_{e}=-\epsilon \kappa^{2}\psi ,$$ (10)

with *κ* being the Debye-Hückel parameter that is related to the EDL thickness, $\lambda_{D}=\kappa^{-1}$. The electric field in Eq. (9) is related to the electrical potential as

E=−∇Φ, (11)

where the electrical potential Φ is given by the sum of the externally imposed electric potential, ϕ, and the electric potential due to the net charge accumulation near the walls, *ψ*

Φ=ϕ+ψ. (12)

These two contributions to the electrical potential are computed using the following equations:

$$\nabla^{2}\phi =0,$$ (13a)

$$\nabla^{2}\psi =-\rho_{e}/\epsilon ,$$ (13b)

with *ϵ* representing the electrical permittivity.

Electro-osmotic flows are investigated in Sec. VI using the OpenFOAM^®^ solver, which follows the method described by Afonso *et al*. (2012). The equations were implemented over *simpleFoam*, which can be used as a steady-state solver for Newtonian incompressible fluids in the OpenFOAM^®^ CFD toolbox. The coupling between pressure and velocity is ensured by the SIMPLE algorithm. The convective terms were discretised using the second-order MINMOD high resolution-scheme, while the diffusive terms were discretised using central differences.

## V. PRESSURE-DRIVEN FLOW

### A. Optimised design in 2D

**FIG. 4.**
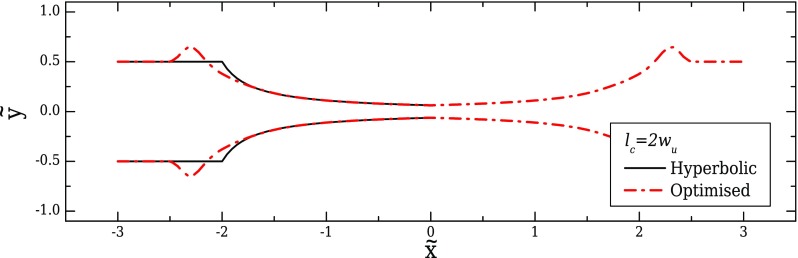
Comparison between the optimised shape (*dashed-dotted line*) and the ideal hyperbolic design (*continuous line*) discussed in Oliveira *et al.* (2007) (2D, CR = 8, *l_c_* = 2*w_u_*, and *l_ε_* = *w_u_*).

In this section, we report the optimisation results for pressure-driven flow in two dimensions considering a constant contraction length, *l_c_* = 2*w_u_*. The shape obtained from the optimisation is presented in Fig. 4 and is compared with the ideal hyperbolic shape, which would be expected for unidimensional flows without velocity gradients in the transverse direction. As already discussed, the ideal hyperbolic shape has been widely used in the past to design converging/diverging geometries. The same general function suggested in Oliveira *et al.* (2007) is used in order to design the walls of the hyperbolic-shaped device, by considering the following expression:

$$|\tilde{y}|=\{\begin{array}{cc} {\left[2\text{CR}(1-|\tilde{x}|\frac{w_{u}-w_{c}}{l_{c}})\right]}^{-1} & \;\text{for}\;|\tilde{x}|\leq n_{1}\\ 1/2 & \;\text{for}\;|\tilde{x}|>n_{1}. \end{array}$$ (14)

**FIG. 5.**
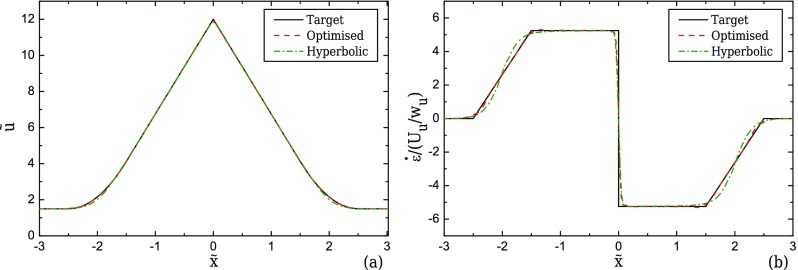
Velocity (a) and strain-rate (b) profiles computed for creeping flow conditions along the centreline of the flow for the optimised geometry and the ideal hyperbolic design (2D, CR = 8, *l_c_* = 2*w_u_*, and *l_ε_* = *w_u_*). The target velocity profile is represented as a *continuous line*.

It can be seen that both designs perform well, both in terms of the velocity and the strain-rate profiles (Figs. 5(a) and 5(b), respectively). The optimised shape exhibits shiftings at the beginning and at the end of the contraction/expansion region (Fig. 4), resulting in a better approximation of the desired strain-rate profile in the transition regions, as shown in Fig. 5(b).

In order to assess the dependence of the optimised solution on the mesh refinement, besides the base mesh M0, a refined mesh M1 was also used (Table I). A very good agreement between the computed velocity profiles is reported, with the maximum deviation in the strain-rate being less than 0.5%.

### B. Contraction length effects

**FIG. 6.**
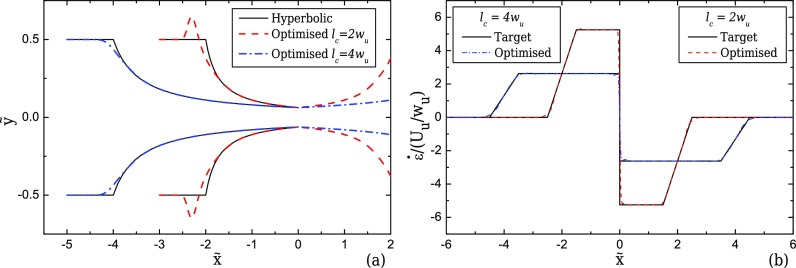
Shapes of the optimised and the ideal hyperbolic devices (a) and strain-rate profiles along the flow centreline for the optimised devices (b) with *l_c_* = 4*w_u_* and *l_c_* = 2*w_u_* (2D, CR = 8, and *l_ε_* = *w_u_*) for creeping flow conditions. Note that in (a) *x* and *y* axes are not to scale.

The choice of the desired contraction length *l_c_* is crucial for the performance and for the final shape of the optimised device. The results obtained show that as the contraction length decreases, the optimisation procedure produces geometries with larger deviations from the hyperbolic shape at the start and the end of the converging/diverging region, as shown in Fig. 6(a). Conversely, the opposite happens as we increase the length of the contraction, where the optimisation procedure predicts optimal shapes approaching the ideal hyperbolic geometry. This finding is particularly important for experimentalists wishing to use the hyperbolic function for designing their microfluidic geometries, or for applications that are especially built for studying specific properties under extensional flow. For example, in studies where a large strain history is required, the hyperbolic shape will in fact perform well, providing a reasonable approximation to the linear velocity profile. However, when it comes to applications where stretch should be quick and in a short length of the device, the use of optimisation for obtaining a more appropriate design with enhanced performance is required.

### C. Channel depth effects

When the devices have low or moderate depth, as is typical in microfluidic platforms, three dimensional effects due to wall interactions need to be taken into account. In such cases, the flow dynamics are different and the optimised shape obtained for 2D flow will not be adequate. In this section, we investigate the effect of aspect ratio on the optimised shape of the geometry considering the same contraction length *l_c_* = 2*w_u_*. Defining the aspect ratio based on the upstream part of the channel as AR = *w_u_*/*d* (where *d* is the depth of the device), we consider the cases of a 3D geometry with a square cross-sectional area in the middle of the contraction/expansion region (AR = 8), another with a square cross-sectional area at the inlet (AR = 1) and two intermediate cases with AR = 2 and AR = 4.

In order to find the designs that will produce the desired constant strain-rate regions along the flow centreline, shape optimisations have been performed considering symmetry conditions along *xy*- and *xz*-centreplanes in order to reduce the cost of the CFD simulations required at every optimisation step. That way, only a quarter of the full geometry was simulated. The target profiles for the 3D cases were constructed by evaluating the maximum velocity from the fully developed velocity profiles upstream and in the middle of the contraction along the centreline (*y* = 0, *z* = 0), using the analytical solution given for each AR (White, 2006):

$$u(y,z)=\frac{12Q}{\pi^{3}ab}\frac{\sum_{i=1,3,..}^{\infty }{\left(-1\right)}^{\frac{i-1}{2}}\left[1-\frac{\text{cosh}(i\pi z/2a)}{\text{cosh}(i\pi b/2a)}\right]\frac{\;\text{cos}(i\pi y/2a)}{i^{3}}}{1-\frac{192a}{\pi^{5}b}\sum_{i=1,3,..}^{\infty }\frac{\text{tanh}(i\pi b/2a)}{i^{5}}},$$ (15)

where *Q* is the flow rate and *a*, *b*, are the half-width and half-depth of the channel cross-section respectively.

**FIG. 7.**
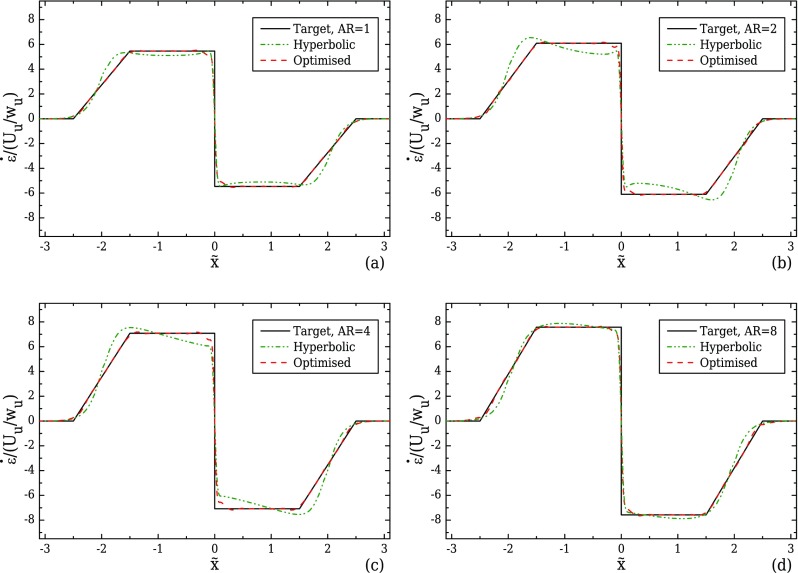
Strain-rate profiles along the flow centreline computed under creeping flow conditions for a geometry with *l_c_* = 2*w_u_*, *l_ε_* = *w_u_*, and CR = 8 and (a) AR = 1, (b) AR = 2, (c) AR = 4, and (d) AR = 8. The optimised shapes are compared with the hyperbolic function.

Initially, the performance of the hyperbolic shape was examined for all aspect ratios. Figure 7 shows the strain-rate profiles along the flow centreline. The hyperbolic geometry does not generally perform as well as for the 2D cases, exhibiting large deviations from the desired target profile, especially for the two intermediate cases of AR = 2 (Fig. 7(b)) and AR = 4 (Fig. 7(c)). For 3D planar channels, characteristic of microfluidics, the varying rectangular cross-section results in velocity profiles that are not necessarily the same in both transverse directions but depend on the local aspect ratio, which explains this deviation. Considering the upstream velocity profiles along the centreplanes *xz* and *xy* for the case of AR = 1, both profiles will be identical since *d* = *w_u_*. However, as the fluid flows towards the middle of the contraction and the width of the channel decreases, the profile on the *xz*-plane will gradually become more flattened than the *xy*-plane profile, reaching a maximum difference in the throat of the contraction/expansion region. A similar but inversed behaviour is found in the case of AR = 8, with the velocity profile in the *xy*-plane exhibiting a more flattened region close to the centreline when compared to the profile in the *xz*-plane in the region upstream of the contraction and identical velocity profiles in both planes at the throat, where *d* = *w_c_*. These gradual transitions of the examined limiting cases (AR = 1 and AR = 8) result in some deviation in the strain-rate profiles shown in Figs. 7(a) and 7(d). These deviations are however more pronounced for the two intermediate cases of AR = 2 and AR = 4, as shown in Figs. 7(b) and 7(c), where the local aspect ratio varies from above one upstream of the contraction to below one at the throat of the contraction/expansion region.

**FIG. 8.**
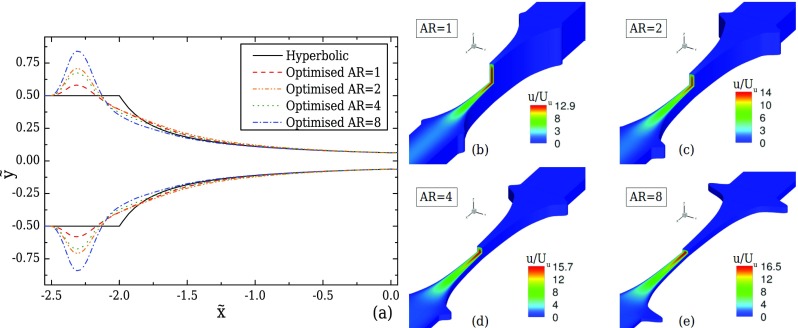
(a) Comparison of the channel boundaries obtained from 3D optimisations for creeping flow conditions for AR = 1, 2, 4, and 8 and the ideal hyperbolic shape when *l_c_* = 2*w_u_*, *l_ε_* = *w_u_*, and CR = 8. (b)–(e) Corresponding contour-plots of the normalised streamwise velocity for each optimised geometry.

The optimised shapes for each aspect ratio are presented and compared with the hyperbolic shapes in Fig. 8(a). Clearly, the boundaries are deformed according to the different flow kinematics in each geometry, exhibiting different sizes in the shiftings of the boundary upstream of the start of the converging region. The maximum shift of the boundary relative to the hyperbolic case is approximately 16% for AR = 1, 42% for AR = 2, 35% when AR = 4 and 68% for AR = 8. More importantly, there are significant differences between the optimised and the hyperbolic shapes in the first third of the contraction, with the differences becoming negligible in the central region $|\tilde{x}|≲1.3$ (cf. Fig. 8). Applying these deformations on the boundaries of the device, the desired strain-rate profiles along the flow centreline are better approximated, as shown in Fig. 7, where all four cases exhibit a maximum deviation of approximately 1%.

**FIG. 9.**
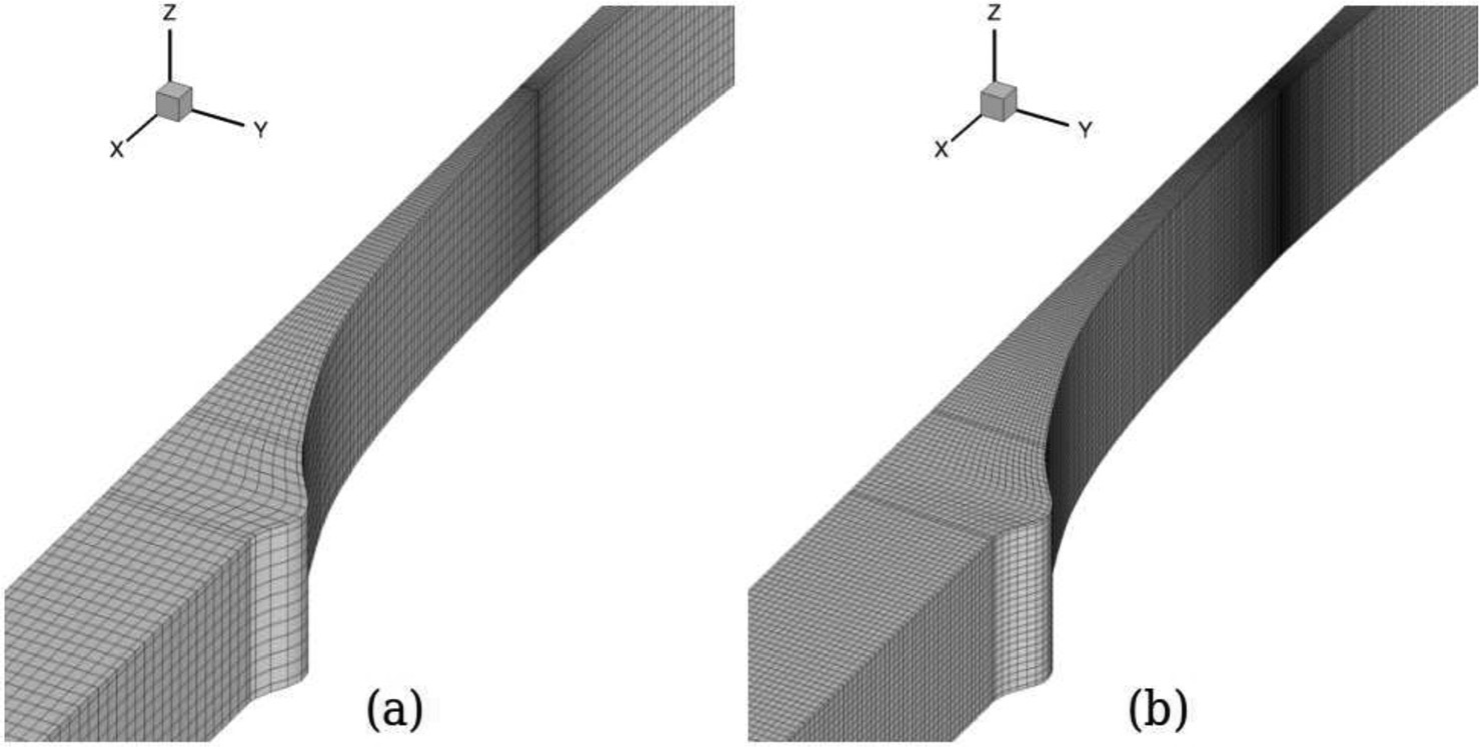
Meshes M0 (a) and M1 (b) for the optimised geometry with *l_c_* = 2*w_u_*, *l_ε_* = *w_u_*, CR = 8, and AR = 1.

As in the 2D case, two meshes were employed for each AR, with mesh M0 being used in the optimisation procedure and mesh M1 for assessing the dependence of the optimised solution on the mesh refinement (cf. Table II). For AR = 1 and AR = 2, the maximum deviation between the two solutions was approximately 1.0%, for AR = 4 was approximately 0.7% and for AR = 8 was approximately 0.5%. Figure 9(a) illustrates the mesh used for obtaining the optimal solution of the design (only a quarter of the geometry is used) for AR = 1, and Fig. 9(b) shows the corresponding refined mesh M1.

### D. Design limits

In this section, we report the operational limits of the 3D configurations presented in Sec. V C. More specifically, the performance of all designs is examined for various Reynolds numbers considering Newtonian fluid flow, using the refined mesh M1. Moreover, the performance of the configuration with AR = 1 is investigated for viscoelastic fluids as a function of the Weissenberg number, under creeping flow conditions.

**FIG. 10.**
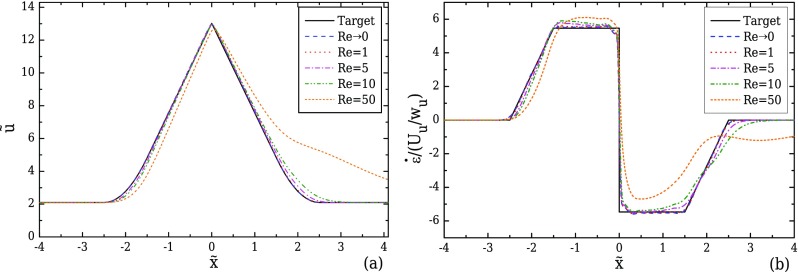
Effect of Re on the velocity (a) and strain-rate (b) profiles computed along the flow centreline, for the optimised geometry with *l_c_* = 2*w_u_*, *l_ε_* = *w_u_*, CR = 8, and AR = 1 (Fig. 8), considering Newtonian fluid flow.

Figure 10(a) shows the effect of Re on the velocity profile along the centreline obtained for a Newtonian fluid in the optimised geometry for AR = 1. For low Re, the geometry optimised under creeping flow conditions performs well, but it is clear that for Re ≳ 5 the kinematics in the device start deviating from the target, affecting noticeably the evolution of the resulting strain-rate profiles, as shown in Fig. 10(b). As mentioned previously, all optimisations were conducted considering creeping flow conditions, where the flow field is symmetric upstream and downstream of the contraction. However, entrance and exit effects on the contraction/expansion region become more prominent as Re is increased, resulting in asymmetric behaviour between the converging and diverging parts of the contraction. It should be noted that for this particular case, flow recirculations are observed downstream of the expansion for Re ≳ 50.

**FIG. 11.**
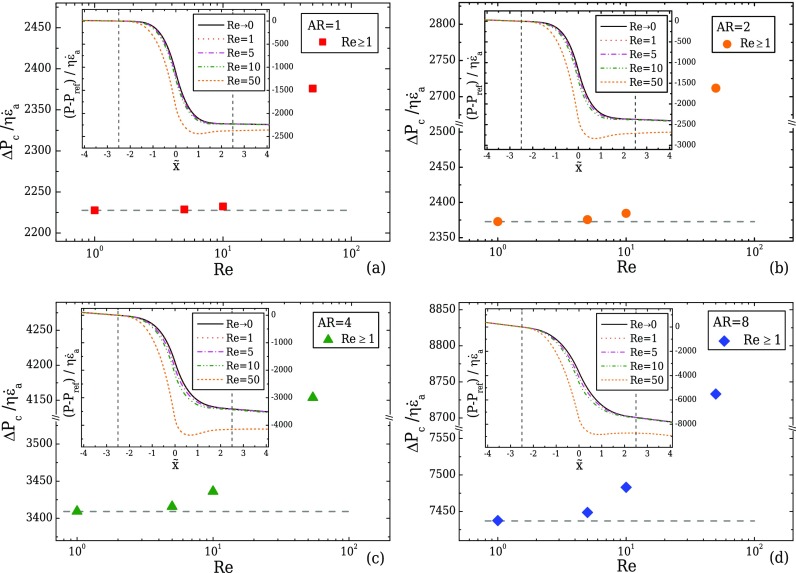
Normalised pressure drop for various Re across the contraction for a geometry with *l_c_* = 2*w_u_*, *l_ε_* = *w_u_*, CR = 8 and (a) AR = 1, (b) AR = 2, (c) AR = 4, and (d) AR = 8, with the *horizontal dashed lines* indicating the normalised pressure drop value under creeping flow conditions. The inset figures in (a)–(d) represent the normalised pressure profiles along the centreline for each case, with the *vertical dashed lines* indicating the start and the end of the transition region of the contraction/expansion.

Figure 11 illustrates the normalised pressure drop, $\Delta P_{c}/(\eta {\dot{\varepsilon }}_{a})$, between the start and the end of the transition region of the contraction/expansion ($-n_{1}-\frac{n_{2}}{2}\leq \tilde{x}\leq n_{1}+\frac{n_{2}}{2}$) for increasing Re numbers, with ${\dot{\varepsilon }}_{a}$ corresponding to the apparent strain-rate evaluated as ${\dot{\varepsilon }}_{a}=(U_{c}-U_{u})/l_{c}=(CR-1)U_{u}/l_{c}$. The inset figures present the normalised pressure profile along the centreline for all Re, calculated based on a reference pressure value, *P*_ref_, taken at the beginning of the transition region ($\tilde{x}=-n_{1}-\frac{n_{2}}{2}$) of each geometry. It can be seen that for Re ≲ 5, the increase in the nominal strain-rate results in an almost linear increase in the pressure drop for all cases. However, for higher Re, this linearity breaks, and the strain-rate along the centreline becomes asymmetric in the two parts of the design, similar to the behaviour observed in Fig. 10 for AR = 1. Note that Re is evaluated using the upstream flow conditions, but its value at the contraction will differ for each of the designs. More specifically, since the Reynolds number reported is based on upstream flow conditions, the Reynolds number at the throat is higher for larger AR, justifying the higher deviation of the normalised pressure drops from the equivalent creeping flow value (*dashed line*) for the shallower designs when Re ≳ 5 (c.f. Figs. 11(c) and 11(d)).

**FIG. 12.**
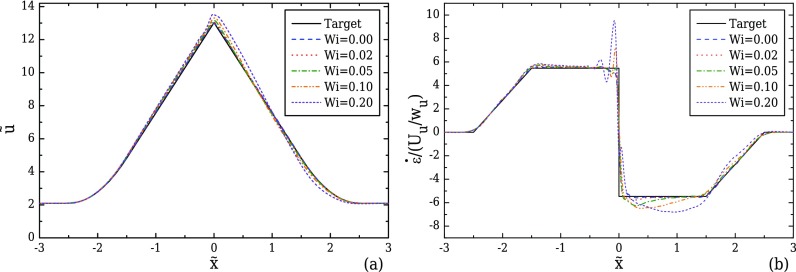
Effect of Wi on the velocity (a) and strain-rate (b) profiles along the flow centreline computed for creeping flow conditions, for the optimised geometry with *l_c_* = 2*w_u_*, *l_ε_* = *w_u_*, CR = 8, and AR = 1 (Fig. 8), for the Oldroyd-B model (*β* = 0.5).

For viscoelastic fluid flows, the effect of elasticity on the velocity field is examined by performing simulations of viscoelastic fluid flows at increasing Wi, using the optimised geometry for AR = 1 under creeping flow conditions. As presented in Sec. IV, we consider two different viscoelastic models, the Oldroyd-B model (*ε* = 0, *β* = 0.5) and the linear form of the PTT model (*ε* = 0.25, *β* = 0.01). Using the Oldroyd-B model, we investigate the influence of elasticity alone as there is no shear-thinning. Figure 12(a) shows that in the converging part of the channel a reasonably good approximation to the linear increase in the velocity profile is achieved for all Wi. However, increasing Wi leads to progressively higher velocity overshoots close to the throat of the converging/diverging region where the maximum velocity is reached. Velocity overshoots in contraction flows of viscoelastic fluids have also been reported by Poole *et al.* (2007) for a UCM fluid, and Oliveira (2003) for a FENE-CR model. This deviation from the target velocity profile is particularly noticeable in the streamwise velocity gradient profiles in Fig. 12(b), with increasing fluctuations near $\tilde{x}=0$ and a clear overshoot for higher Wi. For Wi = 0.20, the strain-rate overshoot deviates approximately 74% from the desired constant value. This overshoot clearly affects the behaviour in the diverging part of the channel, and for Wi ≥ 0.05, the strain-rate profiles can no longer be considered constant. Furthermore, the asymmetric profiles between the converging and the diverging part of the contraction demonstrate that the strain history affects the velocity profile development, indicating that as Wi increases the fluid memory becomes important. For Wi ≤ 0.02, the strain-rate can be considered nearly constant, with a maximum deviation of approximately 5% in the beginning of the diverging region.

**FIG. 13.**
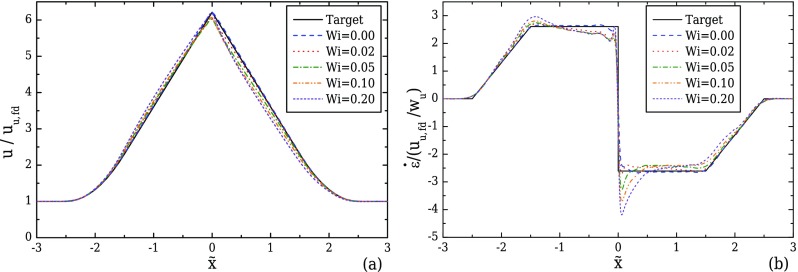
Effect of Wi on the velocity (a) and strain-rate (b) profiles along the flow centreline computed for creeping flow conditions, for the optimised geometry with *l_c_* = 2*w_u_*, *l_ε_* = *w_u_*, and CR = 8 for AR = 1 (Fig. 8), considering a PTT fluid (*ε* = 0.25, *β* = 0.01).

The PTT fluid exhibits a different behaviour for increasing Wi numbers as shown in Fig. 13 as a consequence of the additional shear-thinning behaviour. For the analysis of the velocity profile along the centreline, we normalise the data using the fully developed velocity at the centreline of the upstream channel ($u_{u,\text{fd}}$), since the upstream streamwise velocity profile for a PTT fluid is flattened compared to the Newtonian case due to its shear-thinning behaviour. It can be seen that as the elasticity increases, the velocity profile along the centreline, shown in Fig. 13(a), increases as noted previously for the Oldroyd-B model. However, before the fluid approaches the middle of the contraction the velocity gradient starts to decrease, forming a small overshoot upstream of the diverging part in both the velocity and the strain-rate profiles as a consequence of fluid's elasticity. As the fluid flows through the diverging region, the velocity rapidly decreases to smaller values than the target profile, affecting the development of the strain-rate profile (Fig. 13(b)), where an undershoot is observed at the beginning of the diverging part. Both the overshoot and the undershoot become more pronounced with increasing Wi and these deviations should be taken into account when this type of fluid is used.

**FIG. 14.**
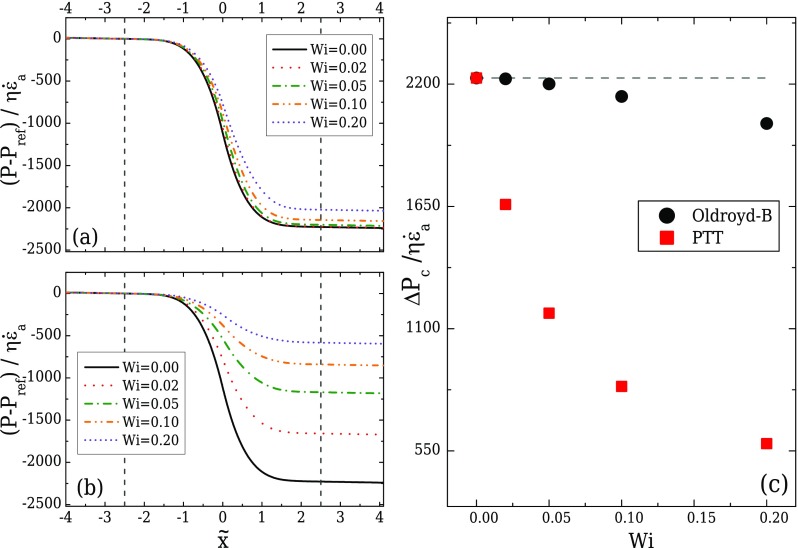
Normalised pressure profile along the centreline for the Oldroyd-B model (a), the PTT model (b) and normalised pressure drop across the contraction/expansion region for both models under creeping flow conditions (c), when the optimised geometry with *l_c_* = 2*w_u_*, *l_ε_* = *w_u_*, CR = 8, and AR = 1 is used. The start and the end of the transition region of the contraction/expansion are indicated by the *vertical dashed lines* in (a) and (b). The *horizontal dashed line* in (c) corresponds to the normalised pressure drop for the Newtonian fluid under creeping flow conditions.

Figures 14(a) and 14(b) present the variation of the normalised pressure profile along the centreline for the Oldroyd-B and the PTT fluids, respectively. It can be seen that both models predict smaller pressure drops across the contraction compared to the Newtonian fluid, with the PTT fluid demonstrating higher differences, due to the shear-thinning behaviour. Moreover, the normalised pressure drop between the start and the end of the transition region is shown in Fig. 14(c), demonstrating the decrease of the pressure drop for increasing Wi. Similar behaviour was reported by Binding *et al*. (2006), for the flow of an Oldroyd-B fluid along a contraction/expansion geometry. In contrast to numerical findings, experimental measurements for viscoelastic fluid flows in contraction geometries demonstrate pressure drop enhancement and an additional flow resistance with the increase in fluid elasticity for Boger fluids (Nigen and Walters, 2002 and Campo-Deaño *et al.*, 2011). This inability of the closed-form viscoelastic models to predict the correct pressure drop is a well known drawback of viscoelastic numerical studies (Owens and Phillips, 2002 and Alves *et al*., 2003b). These constitutive models do not contain sufficient information related to the micro-structure of the polymer chains, and it is believed that possible inclusions at this level will assist in capturing the physics of polymer flows with greater accuracy (Rothenstein and McKinley, 2002).

## VI. ELECTRO-OSMOTIC FLOW

In this section, the optimisation of a converging/diverging channel is considered for electro-osmotic flow. Similarly to what was described in Sec. V for pressure-driven flow, here we consider only the case of $l_{c}=2w_{u},\;l_{\varepsilon }=w_{u}$ for CR = 8. For this case, an additional mesh refinement was used, in order to resolve accurately the flow inside the EDL (cf. Table III). The grading was such that at least 10 cells existed within a distance of 1/*κ* from the walls, where *κ* = 200/*w_c_* was used for optimisation purposes (i.e., an electric double layer which is 100 times thinner than the channel contraction half-width). Moreover, only 2D geometries were considered in the optimisation, although the final optimal shapes were also tested in 3D configurations in order to assess the aspect ratio independence of the centreline velocity.

### A. Optimised design

**FIG. 15.**
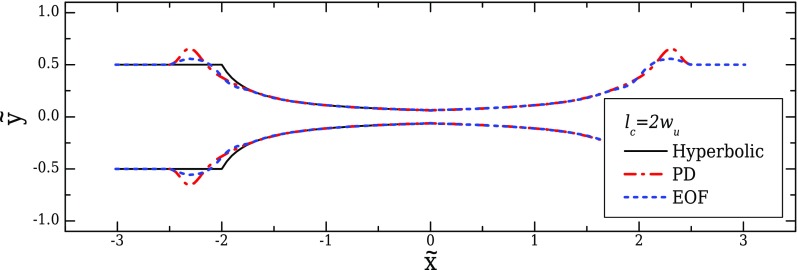
Optimised shapes obtained for pressure-driven flow (PD) and for electro-osmotic flow (EOF), in contrast with the ideal hyperbolic geometry (2D flow, *l_c_* = 2*w_u_*, *l_ε_* = *w_u_*, and CR = 8).

**FIG. 16.**
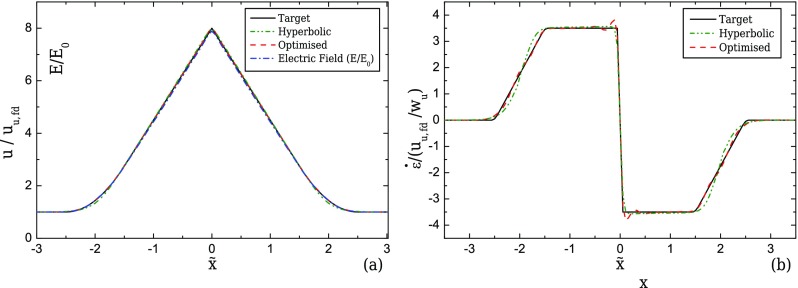
Velocity profiles computed in the optimised geometry and comparison with the computed electric field (a) and the resulting strain-rate (b) profiles along the centreline for EOF. The profiles obtained with EOF using the ideal hyperbolic geometry are also plotted (2D, *l_c_* = 2*w_u_*, *l_ε_* = *w_u_*, and CR = 8).

The optimised shapes and the corresponding flow kinematics along the centreline for EOF are presented in Figs. 15 and 16, respectively. Mesh M0-EOF was used for optimisation purposes, for which the peak strain-rate at the centreline deviates less than 1% from the value on the more refined mesh M1-EOF. The optimised geometries only differ significantly from the ideal hyperbolic shape in the transition regions. This fact demonstrates that the ideal hyperbolic shape is in fact very close to the optimal shape for a region of constant strain-rate and the optimisation procedure is only useful to control (impose) the strain-rate profile at the transition region. The good performance of the hyperbolic shape in electro-osmosis is not surprising, since that shape is the analytical solution for a constant strain-rate region assuming potential flow (Ober *et al.*, 2013). For the conditions of the present work, although the electric field is irrotational, the velocity is not irrotational due to internal pressure gradients and the no-slip conditions at the walls. Therefore, the similitude between the electric field and the velocity field is broken (Cummings *et al.*, 2000 and Santiago, 2001; 2007). However, a quasi-potential flow can still be considered, since the added dynamic pressure due to changes in the cross-sectional area of the channel, which reflects on a velocity variation, is relatively small and the electric double layers are thin. This is proved both by the good performance of the hyperbolic shape, as well as by the quasi-linearity of the electric field (*E*/*E*_0_, where *E*_0_ is the uniform value of the electric field at the inlet/outlet of the channel) along the centreline of the contraction/expansion region, as shown in Fig. 16(a). This last observation further points to the possibility of replacing the velocity variable by the electric field to define the objective functions. Indeed, due to the similitude between both fields, this procedure would lead to a dramatic reduction in the CPU time for the optimisations, since the Navier-Stokes equations coupled to the electric field equations would be replaced by a single Laplace equation to be solved for the electric field. We have not followed this approach because for higher Hencky strains, the internal pressure gradients increase and the similitude between the velocity and the electric field is weaker, leading to differences between electric field and velocity profiles (results not shown). The implemented routine is thus more general, at a cost of a higher computation time.

It is worth to note that having a linear electric field profile at the channel centreline is also a desired feature when considering extensional flows driven by electrophoresis, since electrophoretic motion follows the electric field lines and a constant strain-rate will be imposed. Therefore, it is of no surprise that the electrophoretic extension of long molecules, as *λ*-DNA was already performed in hyperbolic micro-contraction devices (Larson *et al.*, 2006; Randall *et al*., 2006; and Hu *et al*., 2009).

### B. Aspect ratio effect

**FIG. 17.**
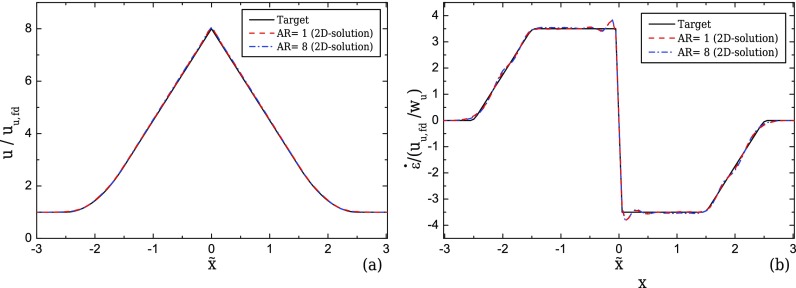
Velocity (a) and strain-rate (b) profiles computed numerically for EOF using the 2D optimised shapes in 3D configurations with *l_c_* = 2*w_u_*, *l_ε_* = *w_u_*, and CR = 8 for AR = 1 and AR = 8.

When the conditions for the similitude between velocity field and electric field are fulfilled, it was demonstrated that the velocity does not depend on the channel depth (Cummings *et al.*, 2000). Hence, the 2D shapes obtained and discussed in Sec. VI A can be generalised to 3D configurations without loss of performance, something that was not possible for the geometries optimised for pressure-driven flow, as shown in Sec. V C. This was numerically confirmed and the results are plotted in Fig. 17(a) for the velocity and in Fig. 17(b) for the strain-rate profiles along the centreline for two 3D configurations, with AR = 8 and AR = 1, corresponding to the maximum and minimum AR examined for the pressure-driven flow cases (Sec. V).

## VII. CONCLUSIONS

We use shape-optimisation numerical procedures to design microfluidic devices that are able to produce specific and well controlled flow kinematics. The design of various converging/diverging geometries with different aspect ratios and a constant contraction ratio (CR = 8) have been optimised to generate a region of constant strain-rate along the centreline, under creeping flow conditions.

In the 2D limit when the contraction region is long enough, the outline of the geometries optimised to produce a region of constant strain-rate approaches an ideal hyperbolic shape. In this case, the optimisation procedure is only useful to control the strain-rate profile of the transition region at the contraction entrance and expansion exit. However, such limits are seldom used in practice in lab-on-a-chip devices, for which the well-known hyperbolic shape is not the most suitable configuration for producing homogeneous extensional flows. As the contraction becomes shorter, entrance and exit effects affect the strain-rate distribution, an issue also observed experimentally by Oliveira *et al.* (2007) and Ober *et al.* (2013). In order to overcome this problem, the optimised geometry exhibits transition regions at the start and at the end of the contraction/expansion, which become larger for short lengths. Even more dramatic is the effect of having 3D planar configurations with close bounding walls as typically used in lab-on-a-chip devices. This introduces a variable aspect ratio along the contraction. The optimisation procedure for a 3D device generated shapes that significantly improved the performance relative to the hyperbolic shape and are unique for each aspect ratio (1 ≤ *AR* ≤ 8). This outcome is important and may be useful as a guideline to help experimentalists better decide upon the appropriate shape to be used.

We showed that all configurations obtained for 1 ≤ AR ≤ 8 perform well up to Re ≈ 5 for Newtonian fluids. Additionally, for the viscoelastic fluids studied using the design of AR = 1, it was demonstrated that when they exhibit significant shear-thinning (PTT model), the optimised configuration for Newtonian fluid flow fails to produce a constant strain-rate along the flow centreline even for low Wi numbers, whereas for the constant viscosity viscoelastic fluid (Oldroyd-B), it can be used accurately in the full converging/diverging region up to Wi = 0.02 (or even higher if we are interested in the converging region alone). Use beyond these limits would require optimisations for the particular fluid/flow condition under consideration.

In contrast to pressure-driven flows, for electro-osmotic flow, the optimised geometries are nearly hyperbolic for both 2D and 3D configurations. The more interesting advantage of EOF in this flow topology is the reduced shear effects, producing a wider region of constant strain-rate around the centreline, as a consequence of the typical developed plug-like velocity profile in electro-osmotic flows.

The geometries optimised in this work, with their inherent simplicity and their ability to generate a wide region of homogeneous strain-rate, can be interesting platforms for studies of cell and droplet deformation, or stretching of single molecules (e.g., DNA, proteins) under uniform controlled extensional flows. In addition, these optimised configurations have the potential to be used for performing measurements of the extensional properties of complex fluids.

## SUPPLEMENTARY MATERIAL

See supplementary material for the effect of the abrupt transition profile at the start of the contraction region and at the end of the expansion region on the shape of the optimised design and its performance.

## Figures and Tables

**TABLE I.** Mesh characteristics for 2D flow simulation when CR = 8 and *l_c_* = 2*w_u_*.

Mesh	*δx_min_*/*w_c_*	*δy_min_*/*w_c_*	#Computational cells
M0	0.045	0.045	4862
M1	0.023	0.023	19448

**TABLE II.** Mesh characteristics for the 3D optimisations for a geometry of *l_c_* = 2*w_u_*, *l_ε_* = *w_u_*, and CR = 8 for AR = 1, 2, 4, and 8.

Mesh	*δx_min_*/*w_c_*	*δy_min_*/*w_c_*	*δz_min_*/*w_c_*	#Computational cells
AR = 1				
M0	0.045	0.045	0.364	53 482
M1	0.023	0.023	0.182	427 856
AR = 2				
M0	0.045	0.045	0.182	53 482
M1	0.023	0.023	0.091	427 856
AR = 4				
M0	0.045	0.045	0.091	53 482
M1	0.023	0.023	0.045	427 856
AR = 8				
M0	0.045	0.045	0.045	53 482
M1	0.023	0.023	0.023	427 856

**TABLE III.** Mesh characteristics for the EOF optimisations for a 2D geometry with CR = 8.

Mesh	*δx_min_*/*w_c_*	*δy_min_*/*w_c_*	#Computational cells
M0-EOF	0.104	0.000488	10 500
M1-EOF	0.052	0.000244	42 000

## APPENDIX: DATA AVAILABILITY

Data files containing the outlines of the optimised shapes presented in this paper are available for download at http://dx.doi.org/10.15129/7928f08f-aacd-4e12-b3b5-a1a1539a7dc1.
